# Spatial accessibility to health facilities among pregnant women with and without exposure to intimate partner violence in Uganda

**DOI:** 10.1186/s12884-023-06084-5

**Published:** 2023-11-03

**Authors:** Ronald Anguzu, Rebekah J. Walker, Kirsten M.M. Beyer, Yuhong Zhou, Harriet M. Babikako, Julia Dickson-Gomez, Laura D. Cassidy

**Affiliations:** 1 Division of Epidemiology and Social Sciences, Institute for Health and Equity, Medical College of Wisconsin, 8701 Watertown Plank Road, Milwaukee, WI 53226 USA; 2 Center for Advancing Population Sciences (CAPS), Medical College of Wisconsin, 8701 Watertown Plank Road, Milwaukee, WI 53226 USA; 3 Division of General Internal Medicine, Department of Medicine, Medical College of Wisconsin, 8701 Watertown Plank Road, Milwaukee, WI 53226 USA; 4 Department of Epidemiology and Biostatistics, School of Public Health, Makerere University, New Mulago Gate Road, Kampala, Uganda

**Keywords:** Spatial accessibility, Intimate Partner Violence during pregnancy, Antenatal Care

## Abstract

**Background:**

Poor physical access to health facilities could increase the likelihood of undetected intimate partner violence (IPV) during pregnancy. We aimed to determine sub-regional differences and associations between spatial accessibility to health facilities and IPV among pregnant women in Uganda.

**Method:**

Weighted cross-sectional analyses were conducted using merged 2016 Uganda Demographic and Health Survey and 2014 Uganda Bureau of Statistics health facility datasets. Our study population were 986 women who self-reported being currently pregnant and responded to IPV items. Outcome was spatial accessibility computed as the near point linear distance [< 5 km (optimal) vs. ≥ 5 km (low)] between women’s enumeration area and health facility according to government reference cutoffs. Primary independent variable (any IPV) was defined as exposure to at least one of physical, emotional, and sexual IPV forms. Logistic regression models were sequentially adjusted for covariates in blocks based on Andersen’s behavioral model of healthcare utilization. Covariates included predisposing (maternal age, parity, residence, partner controlling behavior), enabling (wealth index, occupation, education, economic empowerment, ANC visit frequency), and need (wanted current pregnancy, difficulty getting treatment money, afraid of partner, and accepted partner abuse) factors.

**Results:**

Respondents’ mean age was 26.1 years with ± 9.4 standard deviations (SD), mean number of ANC visits was 3.8 (± 1.5 SD) and 492/986 (49.9%) pregnant women experienced IPV. Median spatial accessibility to the nearest health facility was 4.1 km with interquartile range (IQR) from 0.2 to 329.1 km. Southwestern, and Teso subregions had the highest average percentage of pregnant women experiencing IPV (63.8–66.6%) while Karamoja subregion had the highest median spatial accessibility (7.0 to 9.3 km). In the adjusted analysis, pregnant women exposed to IPV had significantly higher odds of low spatial accessibility to nearest health facilities when compared to pregnant women without IPV exposure after controlling for enabling factors in Model 2 (aOR 1.6; 95%CI 1.2, 2.3) and need factors in Model 3 (aOR 1.5; 95%CI 1.1, 3.8).

**Conclusions:**

Spatial accessibility to health facilities were significantly lower among pregnant women with IPV exposure when compared to those no IPV exposure. Improving proximity to the nearest health facilities with ANC presents an opportunity to intervene among pregnant women experiencing IPV. Focused response and prevention interventions for violence against pregnant women should target enabling and need factors.

## Background

Intimate partner violence (IPV) is a major global health concern defined as any behavior within intimate relationships that results in physical, psychological or sexual harm [1–3]. Sub-Saharan Africa (sSA) suffers from one of the highest rates of IPV during pregnancy, despite being underreported, with on average 15% and in some locations up to 57% of pregnant women reporting IPV [4–8]. In Uganda, 10.6% of pregnant women experience physical forms of IPV and IPV-rates during pregnancy are higher in populations living in rural areas (11%) and the lowest wealth quintile (16.8%) [9]. IPV during pregnancy is associated with poor quality of life, increased risk of unsafe abortion, unintended pregnancy, low self-esteem, lower utilization of maternal health services such as antenatal care (ANC) visits, skilled birth attendance, personal safety concerns, living with HIV, psychoactive substance abuse, and poor mental health [10–18].

Geographical distance to health facilities (spatial accessibility) are supply side factors associated with utilization of health services. Several studies mainly investigated individual-related factors that affect healthcare utilization such as maternal age, educational level, household poverty and cultural beliefs [19–23]. Although some studies have investigated supply factors that affect healthcare utilization such as understaffing and few specialty providers, residing in rural areas, spatial accessibility to health facilities is understudied as supply factor affecting women’s health in Uganda [24–27].

Healthcare facilities provide an opportune setting to routinely screen for IPV and intervene to reduce adverse maternal and child health outcomes. Although Ugandan Clinical Guidelines recommend routine screening in primary healthcare settings for possible IPV, only half of the general population in Uganda have optimal physical access to healthcare, defined as the proportion of people living within a five-kilometer proximity to the nearest health facility [9, 28]. Transport costs, affordability of health services, and lack of psychosocial support are potential barriers to travel to health facilities by many women including women exposed to IPV during pregnancy [29–33]. Referral of women with IPV during pregnancy by healthcare providers and Village Health Teams (VHTs) is also low due to inadequate identification of IPV. In Uganda, few women who experience IPV (10.5%) utilize healthcare and some cultural practices like bride price payment reduces independent decision making to seek ANC [34, 35].

Routine screening for IPV by healthcare providers as well as counseling for and management of complications from IPV are prevention efforts recommended within ANC settings [31, 36, 37]. Although integration of health care is recommended, Uganda’s healthcare provision design has a vertical approach in which both antenatal and mental healthcare are often offered separately. ANC providers primarily focus on obstetric risk screening which increases the likelihood of IPV to go undetected and IPV complications not managed [38]. Little is known about the extent of IPV screening during pregnancy or the feasibility of universal IPV screening [31]. Multi-level strategies have been suggested to recognize and respond to IPV although this has been challenging.

Andersen’s behavioral model of health service utilization helps explain which group of factors explain the relationship between IPV exposure in pregnant women and spatial accessibility to health facilities. *Predisposing factors* refer to factors that predispose an individual’s planned or intended healthcare seeking or utilization. *Enabling factors* are factors that may facilitate health service use or care seeking behavior. *Need factors* refer to actual or perceived views of an individual health and functional needs. This model, enables our assessment of measures of accessibility such as equitable services. We adopted Andersen’s behavioral model of healthcare utilization [39] to select block factors that may explain any differences in the spatial accessibility to health facilities between women with and without exposure to IPV. This model suggests that individual characteristics, namely predisposing, enabling and need factors collectively influence health behavior, particularly healthcare utilization and spatial accessibility may differ among pregnant women exposed to IPV and subsequent health outcomes. To our best knowledge, there is no searchable research describing levels of spatial accessibility to health facilities among women exposed to IPV during pregnancy in Uganda. In order to address this knowledge gap, our study aimed to achieve two objectives. We aimed to (i) examine subregional differences in spatial accessibility to health facilities and IPV exposure among pregnant women, and (ii) determine the association between spatial accessibility to health facilities and IPV among pregnant women in Uganda using Andersen’s behavioral model of healthcare utilization. We hypothesized no differences in spatial accessibility to healthcare facilities between pregnant women with and without exposure to IPV in Uganda.

## Methods

### Study design and site

This was a cross-sectional study of pregnant women at household level during the 2016 Uganda Demographic Health Survey (UDHS) [9]. Uganda has a total population of 42 million people [40]. According to the 2014 National Population and Census Survey, urban areas are defined as cities, municipalities, town councils or town boards or townships with a projected urban population of 9.4 million for mid-2017 [41]. Uganda is geographically divided into fifteen administrative regions with 134 administrative districts that are predominantly rural [9].

### Data sources

The 2016 UDHS and the 2014 Uganda Bureau of Statistics (UBOS) health facility data were analyzed [9]. Research assistants trained by UBOS in collaboration with the Ministry of Health (MoH) conducted data collection between 20th June and 18th December 2016 as described in detail elsewhere [42]. The 2016 UDHS is a two-stage stratified cluster survey and the sampling frame includes all census Enumeration Areas (EA) generated by UBOS. In the first stage, 696 accessible EAs were selected, 162 in urban and 534 in rural areas. In the second stage, a list of households in each EA was used to select households to be surveyed, with women of reproductive age being eligible to participate. Each EA is a geographical area containing an average of 130 households. Two data files from the 2016 UDHS were used, namely (i) the individual survey and (ii) geographic covariate datasets. The survey dataset contains respondent’s socio-demographic characteristics and domestic violence items. Pregnant women with or without exposure to IPV during pregnancy were identified using the IPV and pregnancy status items selected from the Domestic Violence Module. The three forms of IPV measured in the 2016 UDHS were physical, sexual and emotional IPV [9]. Geographic locations of each woman’s residence were estimated using the centroid representing their EA. The geographic dataset contains EA centroid locations as latitude and longitude coordinates.

The 2014 UBOS dataset contains the following variables, namely, public health facility names, their health center (HC) levels, as well as their subregion, subcounty, and district. The variables also included the geographical coordinates of the public health facilities. In Uganda, ANC is facility-based and provided at HC III, HC IV and hospital levels [43]. We merged the 2016 UDHS and 2014 UBOS health facility datasets. Based on their coordinates, we georeferenced all the health facilities at HC IV level into points, to which the nearest distance from each EA/cluster point was calculated in ArcGIS v16.0.

### Study population and sampling procedure

**Fig. 1:**
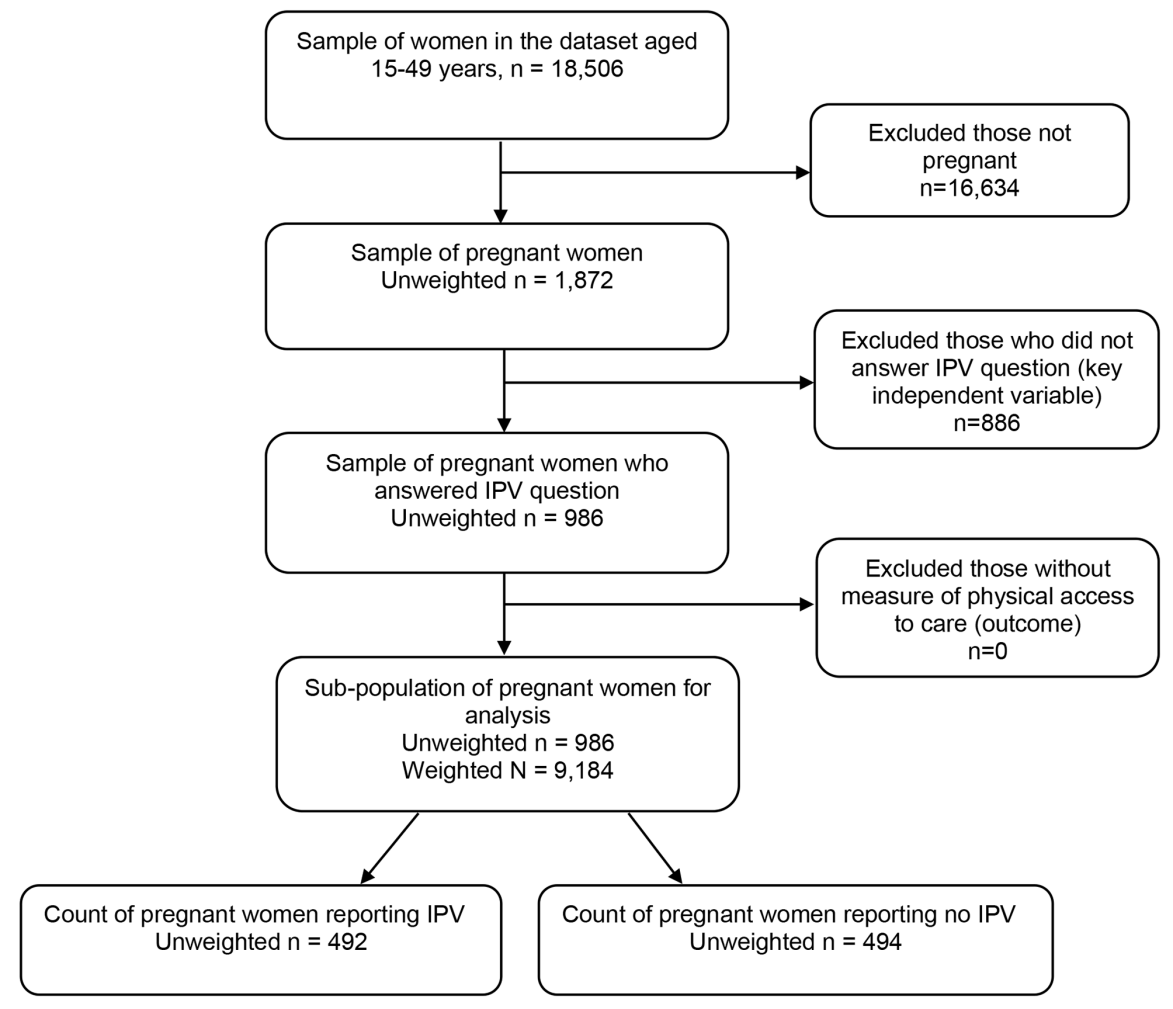
Flow chart showing creation of sample of pregnant women in Uganda ages 15–49 with information on exposure to IPV and spatial accessibility to ANC services

Figure 1 shows the selection criteria for our study population. The UDHS 2016 individual recoded dataset contained a sampling frame of women who were either pregnant or not. Our study sample included any woman who: (i) self-reported being pregnant and (ii) responded with ‘yes’ or ‘no’ to the total IPV items. Generation of the composite IPV variable is described in the sub-section below. Observations were excluded if women were not pregnant or when the items measuring total IPV, or spatial accessibility were missing. Out of 18,506 women in our sampling from the 2016 UDHS individual dataset, we excluded participants who self-reported not being pregnant (n = 16,634) and who did not answer IPV questions (n = 886). The final study sub-population sample size was 986 pregnant women with and without IPV for statistical analysis (Fig. 1).

### Study measures

Outcome variable was spatial accessibility to healthcare facilities by pregnant women with and without exposure to IPV defined as the physical distance between health facilities and participants’ residence (census EA). Spatial accessibility was measured using ArcGIS v16.0 software as the near point linear distance in meters between EA centroids/clusters and the nearest health facility. Spatial accessibility was categorized as ≥ 5 km (low) or < 5 km (optimal) accessibility based on cutoffs described in prior literature [44–47].

#### Measure of IPV exposure

Primary independent variable was exposure to IPV during pregnancy. This study defined ‘partner’ as husband or partner in cohabiting unions. Similar coding was used from prior studies for total IPV, physical IPV and sexual IPV [48]. The Domestic Violence module in 2016 UDHS contains IPV items adopted from the revised Conflict Tactics Scale [49].

For **physical IPV**, women were asked: *In the past 12 months, did your husband/partner (current or last) ever; “push you, shake or throw something at you”, “slap you”*, *“fist punch or hit you with something harmful”*, *“kick or drag you”, “strangle or burn you”, “threaten you with knife/gun or other weapon”* and *“arm twist or hair pulled”.* The 2016 UDHS dataset contained two composite variables namely; (i) *“any less severe or moderate violence”* generated from items and (ii) *“any severe violence”.* These two composite variables; “any severe” and “less severe or moderate” violence were merged into a dichotomous variable “physical IPV in the last 12 months” (No = never experienced physical violence, Yes = experienced physical violence).

Regarding **emotional IPV** items, women were asked: In the past 12 months, did your husband/partner (current or last); *“humiliate you”, “threaten you with harm”, “insult or made you feel bad”.* These three variables were merged into a dichotomous variable for emotional IPV in the last 12 months (No = never experienced emotional violence, Yes = experienced emotional violence).

For **sexual IPV**, women were asked the following questions: In the past 12 months, did your husband/partner (current or past) ever *physically force you into unwanted sex with him*?, *force you into other unwanted sexual acts*? and *physically forced you to perform sexual acts when you did not want to*? In 2016 UDHS, binary (yes/no) responses to these three items were used to compute a composite variable; sexual IPV in the last 12 months (No = never experienced sexual violence and Yes = experienced sexual violence).

#### Creation of any IPV exposure variable

Any IPV form experienced in the last 12 months preceding the survey was created by combining physical, emotional, and sexual IPV variables. Women who reported experiencing either physical, emotional and/or sexual violence were coded 1 (Yes) while those who reported no to all three forms were coded as 0 (No). Andersen’s behavioral model of healthcare utilization informed selection of covariates and were categorized into three blocks: predisposing, enabling and need factors [39] as described below.

#### Predisposing factors

Women’s age and parity (number of children ever born) were continuous variables. Residence was dichotomized into urban and rural. Partner controlling issues or behavior was an existing continuous variable in the DHS dataset computed from three categorical items that asked whether their partners (i) were jealous if respondents talked with other men; (ii) accused them of being unfaithful; (iii) did not permit them to meet female friends; (iv) tried to limit the respondents’ contact with family and (v) insisted on knowing where the respondent was. These items all had binary responses (0 = no/1 = yes). Partner controlling issues in UDHS was a continuous variable computed as a summation of ‘yes’ and ‘no’ responses. In this study, we categorized partner controlling issues into a binary variable (No = 0/Yes = 1 or more controlling behaviors).

#### Enabling factors

Household wealth index is a quintile measure of household poverty generated by UDHS ranging from 1 (poorest) to 5 (richest) [50]. In the current study, we described household wealth index into poorest, poorer, middle, richer, and richest categories. Occupation was categorized into agricultural, non-agricultural, and unemployed. The level of formal education was categorized into no education, primary, secondary, and above secondary while number of ANC visits was a continuous variable. Economic empowerment was a composite categorical variable (No/Yes), generated from two items that asked whether a title deed on land was (i) owned by the respondent only; (No = Does not own land or title deed/Yes = owned land/title deed alone) and (ii) owned jointly (No = neither alone or jointly/Yes = either alone or jointly with a partner). This was recoded as a binary variable (0 = land/title deed not owned by respondent or jointly/Yes = land or title deed owned by respondent either alone or jointly).

#### Need factors

In UDHS, the variable ‘*afraid of her partner’* was categorized as 0 = Never afraid, 1 = sometimes afraid and 2 = most of the time afraid. We recoded “*afraid of partner*” into a binary variable; No = never afraid/Yes = sometimes or most of the time afraid. Acceptance of partner abuse was generated from the variable ‘attitudes accepting violence’ was a composite categorical variable (No/Yes) generated from five binary items. Respondents were asked whether beating is accepted if a wife (i) goes out without telling husband (No/Yes), (ii) neglects the children (No/Yes), (iii) argues with husband (No/Yes), (iv) Refuses to have sex with husband (No/Yes) and (v) burns the food (No/Yes). Overall variable ‘accepted partner abuse’ was a composite categorical variable computed as acceptation of physical violence for any reason (0 = No to items i to v or 1 = Yes to items i to v). Wanted current pregnancy was generated using the categorical variable in 2016 UDHS that assessed whether the respondent wanted their last child. Item responses were (i) wanted then, (ii) wanted later and (iii) not at all. We generated a binary variable for ‘wanted current pregnancy’ (No/Yes) i.e. “No” current pregnancy wanted later or not at all and “Yes” were current pregnancies wanted then. The variable ‘difficulty getting treatment money’ was generated from the item ‘problem getting medical help or money needed for treatment’ and categorized into the responses; (i) ‘problem’ getting treatment money, and (ii) ‘no problem’ getting treatment money.

### Statistical analysis

We conducted the following set of analyses. First, descriptive statistics for characteristics of pregnant women with and without IPV exposure were computed, using means (± SD) and frequencies (%). Chi-square tests were run to test differences between women with and without IPV during pregnancy for categorical covariates. For the continuous variables, non-parametric equality-of-medians test, and corresponding inter-quartile range (IQR) compared median ANC visits and parity by the key independent variable (IPV during pregnancy). We tested and found no collinearity between covariates on the outcome of spatial accessibility. Secondly, unadjusted logistic regression models were run to test for associations between IPV during pregnancy and of each covariate against spatial accessibility. Thirdly, interaction terms were run to test if the relationship between IPV during pregnancy and spatial accessibility differed by rural/urban residence. The interaction term was not significant and thus stratified analyses were not conducted. Fourth, sequential adjusted logistic regression models were run to examine the independent association between IPV during pregnancy and spatial accessibility to health facilities using Andersen’s behavioral model of healthcare utilization as follows: the first adjusted model controlled for predisposing factors (Model 1). The second controlled for enabling factors (Model 2). The third model controlled for need factors (Model 3). All statistical analyses were conducted in Stata/MPversion17.0. We used the Jenks classification method in ArcGIS v16.0 to map the geospatial distribution, and spatial accessibility between clusters and health facilities, as well as the average percentage of pregnant women with IPV exposure at sub-region level respectively. Statistical significance was determined at *p* < 0.05. Survey weights were applied to all analyses based on the UDHS survey design to account for complex survey sampling. Survey commands were run in Stata by applying probability/sampling weight (*pweight*), primary sampling unit (*psu*) and stratification used in the sample design (*strata*). Sampling weights (*pweight)* were computed using the domestic violence weight as described in the DHS methodology. Strata with a single sampling unit were treated as certainty units.

## Results

**Fig. 2:**
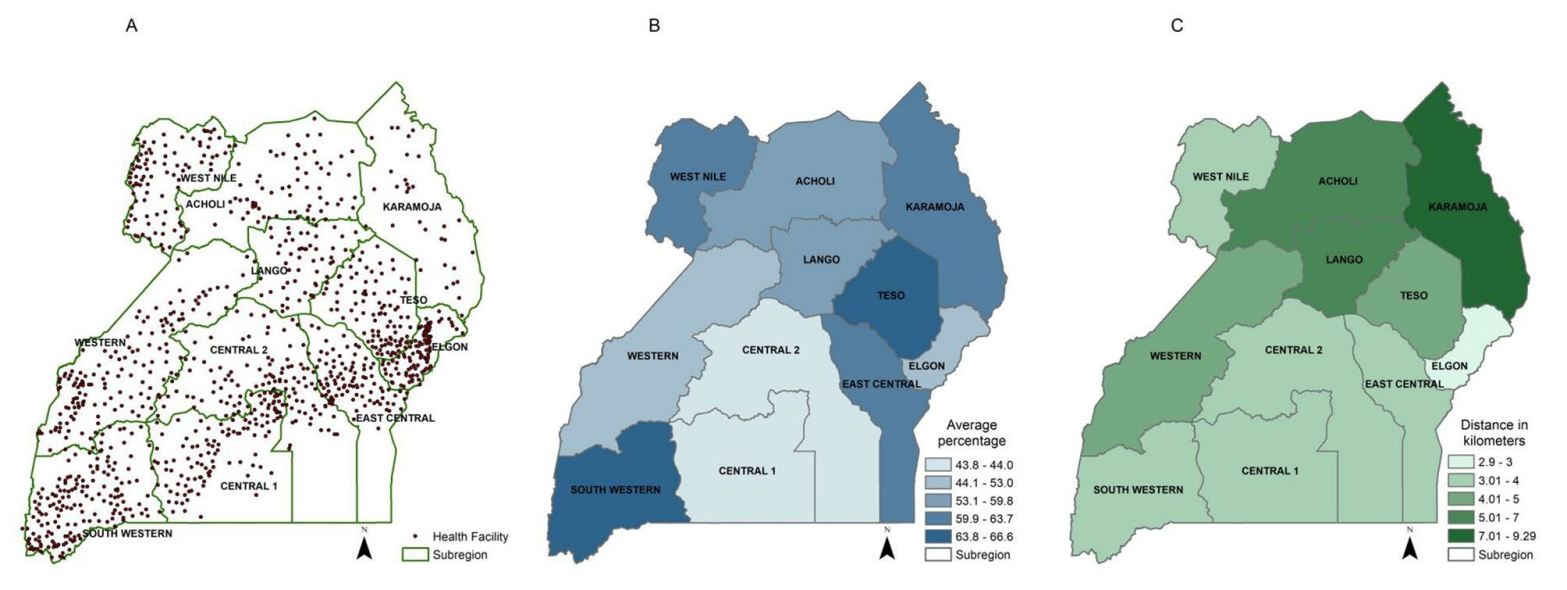
The geospatial distribution of public health facilities, average percentage of pregnant women exposed to intimate partner violence, and spatial accessibility to public health facilities. **A** Geospatial location of public health facilities in Uganda. **B** Average percent of pregnant women experiencing intimate partner violence by sub-region in Uganda. **C** Median distance between clusters and nearest health facilities by sub-regions in Uganda

Mean age of all pregnant women was 26.1 years (SD ± 6.4), mean number of children ever born was 2.7 (SD ± 2.5) and mean number of ANC visits was 3.8 (SD ± 1.5). IPV was experienced by 492/986 women (49.9%) during pregnancy. Overall, 531/986 respondents (53.8%) lived ≥ 5 km from their nearest health facility with a median distance of 4.1 km [IQR 0.21, 329.1] (Table 1). Women with IPV during pregnancy had low spatial accessibility to health facilities (median 4.6 km; IQR 0.42, 329.1) compared to those who did not (median 3.9 km; IQR 0.2, 329.1). Among all respondents, 708/986 (71.8%) had partners with controlling behaviors, 590/986 (59.8%) were not economically empowered, and 526/986 (53.4%) accepted partner abuse. Figure 2 showed the average percentage of pregnant women experiencing IPV and the nearest distance between clusters of respondents’ residence and health facilities at the subregion level. Southwestern, Teso and West Nile subregions have the highest average percentage of pregnant women experiencing IPV ranging from 63.8 to 66.6% while Karamoja region has the highest median distance to nearest health facilities ranging from 7.0 to 9.3 km region (Fig. 2).

**Table 1:** Descriptive characteristics of pregnant women in Uganda with IPV and spatial accessibility data, N = 986

		Any IPV exposure during pregnancy		
		No	Yes	**p-value**
		n(%)	n(%)	
		494 (50.1)	492 (49.9)	
Spatial accessibility, median (IQR)*	4.1 (0.21, 329.1)	3.9 (0.2, 329.1)	4.6 (0.4, 329.1)	0.148
Distance to nearest health facility				
<5 km (optimal)	455 (46.2)	255 (51.6)	200 (40.6)	0.001
≥5 km (low)	531 (53.8)	239 (48.4)	292 (59.4)	
Parity, mean (SD)^a^	2.7 (2.5)	2.5 (2.4)	2.9 (2.4)	< 0.001
Number of ANC visits, mean (SD)	3.8 (1.5)	3.8 (1.2)	3.7 (1.8)	0.679
Maternal age in years, mean (SD)	26.1 (6.4)	25.5 (6.2)	26.5 (6.6)	0.009
Maternal age group, years				0.031
>35	107 (10.9)	42 (8.5)	65 (13.2)	
25–34	409 (41.5)	202 (40.9)	207 (42.1)	
15–24	470 (47.7)	250 (50.6)	220 (44.7)	
Residence				0.015
Urban	182 (18.5)	106 (21.5)	76 (15.5)	
Rural	804 (81.5)	388 (78.5)	416 (84.6)	
Level of formal education				
No education	102 (10.3)	43 (8.7)	59 (11.9)	0.002
Primary	625 (63.4)	304 (61.5)	321 (65.1)	
Secondary	202 (20.5)	106 (21.5)	96 (19.5)	
>Secondary	57 (5.8)	41 (8.3)	16 (3.5)	
Occupation				0.166
Agricultural	433 (43.9)	203 (41.1)	230 (46.8)	
Non-Agricultural	379 (38.4)	196 (39.7)	183 (37.1)	
Unemployed	174 (17.7)	95 (19.2)	79 (16.1)	
Household wealth index				< 0.001
Poorest	257 (26.1)	101 (20.5)	156 (31.7)	
Poorer	223 (22.6)	111 (22.5)	112 (22.8)	
Middle	194 (19.7)	94 (19.0)	100 (20.2)	
Richer	165 (16.7)	89 (18.0)	76 (15.5)	
Richest	147 (14.9)	99 (20.0)	48 (9.8)	
Economic empowerment				0.222
No	590 (59.8)	305 (61.7)	285 (57.9)	
Yes	396 (40.2)	189 (38.3)	207 (42.1)	
Decisions on household expenditure ^b^				0.047
Woman not involved	55 (10.2)	22 (7.7)	33 (12.9)	
Woman involved	486 (89.8)	263 (92.3)	223 (87.1)	
Accepted partner abuse				< 0.001
No	460 (46.7)	259 (52.4)	201 (40.8)	
Yes	526 (53.3)	235 (47.6)	291 (59.2)	
Controlling partner behavior				< 0.001
No	278 (28.2)	211 (42.7)	67 (13.6)	
Yes	708 (71.8)	283 (57.3)	425 (86.4)	
Difficulty getting needed treatment money				0.001
No problem	567 (57.6)	311 (62.9)	256 (52.1)	
Problem	419 (42.4)	183 (37.1)	236 (47.9)	
Afraid of partner				< 0.001
No	545 (55.3)	354 (71.6)	191 (38.8)	
Yes	441 (44.7)	140 (28.4)	301 (61.2)	
Current pregnancy wanted				< 0.001
No	410 (41.6)	178 (35.1)	232 (47.2)	
Yes	576 (58.4)	316 (63.9)	260 (52.8)	

^a^ n = 680, ^b^ n = 541, *_X_1,000 m (1 km = 1,000 m)

Low spatial accessibility was statistically significantly higher among pregnant women who experienced any IPV when compared to those without IPV exposure [292/492 (59.4%) vs. 239/494 (48.4%), p < 0.001] (Table 1). Women with IPV exposure during pregnancy were more likely to live in rural areas compared to urban areas [804/986 (81.5%) vs. 182/986 (18.5%), p = 0.015], and reside in the poorest households, [156/492 (31.7%)] compared to the richest households [48/492 (9.8%)]. More pregnant women who experienced IPV were young aged 15–24 years [220/492 (44.7%)] compared to those over 35 years of age [65/492 (13.2%)].

Unadjusted logistic regression analysis identified factors that were significantly associated with spatial accessibility to health facilities (Table 2). Pregnant women who experienced IPV had significantly higher odds of low spatial accessibility to health facilities when compared to those without IPV [crude Odds Ratio (cOR) 1.2; 95%CI 1.1, 1.5]. Adjusted logistic regression analyses are shown in Models 1 to 3 (Table 2). Exposure to IPV during pregnancy was significantly associated with low spatial accessibility to nearest health facilities when compared to pregnant women without IPV exposure after controlling for enabling factors in Model 2 (aOR 1.6; 95%CI 1.2, 2.3) and need factors in Model 3 (aOR 1.5; 95%CI 1.1, 3.8). After controlling for predisposing factors, however, in Model 1, the relationship of spatial accessibility to nearest health facilities did not differ significantly between women experiencing IPV during pregnancy and those without any IPV exposure (aOR 1.1; 95%CI 0.8, 1.6).

**Table 2:** Logistic regression models testing spatial accessibility to health facilities among pregnant women with and without IPV in Uganda, N = 986

	Unadjusted model	Adjusted models		
		Model 1	Model 2	Model 3
**Characteristics**	cOR (95%CI)	aOR (95%CI)	aOR (95%CI)	aOR (95%CI)
Any IPV				
No	Ref	Ref	Ref	Ref
Yes	1.2 (1.1, 1.5)	1.1 (0.8, 1.6)	1.6 (1.2, 2.3)	1.5 (1.1, 3.8)
**Predisposing factors**				
Maternal age	1.1 (0.9, 1.2)	0.8 (0.7, 0.9)		
Parity	1.4 (1.2, 1.8)	1.3 (1.1, 1.7)		
Residence				
Urban	Ref	Ref		
Rural	2.5 (1.4, 4.5)	2.9 (1.5, 6.1)		
Partner controlling behavior				
No	Ref	Ref		
Yes	0.9 (0.7, 1.1)	0.8 (0.5, 1.2)		
**Enabling factors**				
Household wealth index				
Richest	Ref		Ref	
Richer	1.7 (0.8, 3.4)		1.8 (0.8, 3.9)	
Middle	1.9 (0.9, 3.7)		1.5 (0.6, 3.4)	
Poorer	2.5 (1.3, 5.1)		2.6 (1.1, 5.8)	
Poorest	3.9 (1.9, 7.9)		3.2 (1.4, 7.6)	
Occupation				
Agricultural	Ref		Ref	
Non-Agricultural	0.7 (0.5, 0.9)		1.1 (0.6, 1.6)	
Unemployed	0.9 (0.6, 1.4)		1.7 (0.9, 2.9)	
Level of formal education				
>Secondary	Ref		Ref	
Secondary	0.9 (0.4, 2.2)		0.5 (0.2, 1.3)	
Primary	1.5 (0.6, 3.4)		0.6 (0.2, 1.6)	
No education	2.2 (0.9, 5.2)		0.9 (0.3, 2.7)	
Economic empowerment				
No	Ref		Ref	
Yes	1.5 (1.1, 1.9)		1.2 (0.8, 1.8)	
Number of ANC visits	0.8 (0.6, 0.9)		0.6 (0.4, 0.8)	
**Need factors**				
Wanted current pregnancy				
No	Ref			Ref
Yes	0.6 (0.3, 1.4)			0.9 (0.7, 1.4)
Difficulty getting treatment money				
No problem	Ref			Ref
Problem	1.5 (1.2, 1.9)			1.3 (0.9, 1.9)
Afraid of partner				
No	Ref			Ref
Yes	1.1 (0.9, 1.4)			1.1 (0.7, 1.5)
Accepted partner abuse				
No	Ref			Ref
Yes	1.1 (0.9, 1.5)			1.3 (0.9, 1.9)

*Model 1 controlled for maternal age, parity, rural/urban residence, and partner controlling behavior. Model 2 controlled for household wealth index, women’s occupation, level of formal education, and economic empowerment. Model 3 controlled for wanted current pregnancy, difficulty getting treatment money, being afraid of partner, and acceptance of partner abuse.*

## Discussion

To our best knowledge, this is the first study to apply a theory-based model, Andersen’s behavioral model of healthcare utilization to examine whether spatial accessibility to health facilities and IPV exposure among pregnant women in Uganda are independently associated. Our study revealed that the likelihood of lower spatial accessibility to health facilities in pregnant women exposed to IPV when compared to their counterparts without IPV was independently associated after controlling for predisposing and enabling factors. This study is unique in the application of a combination of geo-spatial analysis techniques and logistic regression analyses to explore the relationship between IPV during pregnancy and physical distance to the nearest health facility. Prior research has shown that maternal and child health (MCH) care is generally difficult to physically access in low and middle income countries [51, 52].

Increasing physical access to health facilities providing ANC could provide opportunities to intervene in pregnant women experiencing IPV in order to improve maternal and child health outcomes. Currently, the Government of Uganda aims to consolidate health infrastructure by strengthening health systems through construction and ensuring functionality of new health facilities [53]. This policy effort is intended to support universal health coverage by increasing the proportion of people with physical access to health facilities. Prior research shows that the proportion of public hospitals in Uganda has decreased over time when compared to private-for-profit and private, not-for-profit hospitals [53]. Our study included public facilities at HC III and IV levels only. HCIV’s are considered higher-level facilities because they provide comprehensive Emergency, Obstetric, and Newborn Care (CEmONC) services which are targeted health facility-based interventions aimed at reducing maternal mortality [54]. However, poor health facility distribution, type of health facility ownership, level of specialty, and functionality may lead to inequity in physical access to facilities and services used [55]. Therefore, several considerations need to be accounted for when setting up new health infrastructure aimed at improving physical access to health facilities. Among them include population-demand for specific health services. This may partly explain the sub-optimal physical accessibility by pregnant women exposed to IPV because the proportion of HCIVs are fewer than lower-level facilities [53]. Our study revealed that pregnant women from the poorer households, less educated, and rural dwellers had longer distance to public health facilities, despite experiencing high levels of IPV. Conversely, economically empowered women had optimal physical access to public health facilities. It is worth noting that distance to health facilities is a social determinant that influences travel costs and means of transport hence contributing to delays or travel time to health facilities [56–59].

Physical distance between households and health units is an important dimension of access to healthcare, that to our best knowledge, has not been evaluated in the context of pregnant populations who have experienced IPV in Uganda [60]. Distance to health facilities may have negative consequences on an individual’s care seeking behavior. For example, IPV is generally under-reported but pregnant women experiencing IPV are more likely to report abuse to health workers than to local councils, police or to seek social support and legal proceedings [61]. Despite paucity of prior research that examined the relationship between IPV and low spatial accessibility, ANC attendees perceive longer distances to health facilities as a barrier to service use [62, 63]. It is noteworthy that with longer distances to health facilities, prior research showed that pregnant women experiencing IPV may reduce their likelihood for abuse disclosure [64] hence subsequent non-detection by health workers. Long walking distances, time and costs of travel are some of the reasons highlighted for inability to report partner abuse to child and family protection units in police [65], and lower the likelihood of facility-based MCH-uptake [66]. One qualitative study showed that adolescent girls do not report IPV to healthcare providers because they would have to travel longer distances to receive medical-check-ups in ANC clinics [65]. Therefore, our main finding underscores the public health importance of increasing the number functional health facilities in areas with poor spatial accessibility to nearest health facilities in Uganda. Such deliberate policy and collaborative efforts to increase health facility infrastructure may address transport challenges, as well as increasing availability of health facilities providing CEmONC. This could reduce limitations of physically accessing health facilities for routine ANC by women with IPV during pregnancy. We posit that greater impact on improved maternal and child health outcomes may be achieved among pregnant populations in Uganda from the poorest households, and no economic empowerment.

Another unique aspect of our study is that by investigating block factors adapted from Andersen’s behavioral model, we discovered that enabling and need factors may be targeted to provide focused interventions to address the barrier of low spatial accessibility to nearest health facilities among pregnant women especially those exposed to IPV. Need factors that our study modelled included whether their current pregnancy was wanted, difficulty in getting treatment money, being afraid of their partners, and acceptance of partners’ abuse. Our theory-informed findings are important because need factors suggest socio-economic needs, and vulnerability which are known to increase the risk of IPV. We posit that future socio-behavioral interventions may change women’s perceptions of long distances to the nearest health facilities as a barrier [26]. Financial incentives and income generation-based interventions may address need factors. These include supporting an individual’s lack of money to pay for ANC like paying clinic user fees (mandatory pre-consultation costs) [67], and introduction of the proposed health insurance scheme in Uganda [68, 69] could lower financial barriers which could prevent retaliatory economic abuse [70] by women’s spouses. Such interventions would complement current efforts by the MoH in Uganda to increase the number of functional health facilities, particularly in rural communities. Psycho-social interventions may provide emotional support to IPV survivors who are afraid of their intimate partners perpetrating abuse [71–74] especially when concerns of safety or life threats arise.

Similarly, addressing enabling factors such as household wealth, employment, and education of the girl child may alleviate poor spatial accessibility to health facilities among pregnant women experiencing IPV. Income generating activities at household level may promote economic empowerment and financial support among women experiencing IPV. Clinical implications of our findings underscore the importance of including patient assessments of these need factors in primary care settings such as ANC [75] including improving provider awareness and screening practices for IPV in health facilities [75] and the range of specialty care offered [76] especially in settings with least spatial accessibility in Uganda. Capital investments in constructing more health facilities or infrastructure in sub-regions with longest median distances to health facilities may improve spatial accessibility to health facilities by pregnant women and likely have broader societal impact on improving maternal and child outcomes in the disadvantaged subregions of Uganda.

### Study strengths and limitations

The current study applied survey weights to compute nationally representative estimates that are generalizable to pregnant women in Uganda. Our study, however, also had some limitations especially related to our estimation of spatial accessibility to health facilities. First, we only included and analyzed public and private-not-for-profit health facilities at HC IV level. Private-for-profit health facilities, and hospitals regardless of ownership were not available in the UBOS dataset. Therefore, it is possible that the poor spatial accessibility to health facilities measured is an over-estimation. Second, spatial accessibility was measured as the nearest, straight-line distance between EA/clusters representing households and health facilities. Under-estimation of the physical distance is likely, especially because Euclidean distances does not account for the terrain (mountainous or flatlands) and streets, roads, or potential pathways available which could provide actual distances. Third, generalizing our findings on spatial accessibility to hospitals and private-for-profit facilities should be avoided. Fourth, this was a cross-sectional study; hence, temporal causal inferences between spatial accessibility and IPV exposure during pregnancy cannot be made. Fifth, the percentage of IPV among pregnant women is higher than the prevalence of IPV in the general population of pregnant women in Uganda. This may be explained by our selection criteria of our study population that included only currently pregnant women who responded to IPV items in the 2016 UDHS survey. Lastly, IQR for spatial accessibility reported in Table 1 were wide, suggestive of skewness. For the current study however, we analyzed spatial accessibility to health facilities as a binary categorical variable.

## Conclusion

Pregnant women experiencing IPV were more likely to have low spatial accessibility to health facilities. The need to improve spatial accessibility to health facilities is underscored in order to provide opportunities to intervene among pregnant women experiencing IPV. Prevention and response strategies aimed at addressing violence perpetrated against pregnant women should target enabling and need factors.

## Data Availability

The datasets generated and/or analysed during the current study are available in the Demographic Health Surveys (DHS) Program repository, https://dhsprogram.com/data/available-datasets.cfm.

## Abbreviations

ANC: Antenatal Care
EA: Enumeration Areas
HC: Health Center
IPV: Intimate Partner Violence
IQR: Interquartile Range
IRB: Institutional Review Board
SSA: Sub-Saharan Africa
TASO: The AIDS Support Organization
UBOS: Uganda Bureau of Statistics
UDHS: Uganda Demographic Health Survey
VHT: Village Health Teams
OR: odds ratio
CI: confidence interval
